# Elimination of Bimodal Size in InAs/GaAs Quantum Dots for Preparation of 1.3-μm Quantum Dot Lasers

**DOI:** 10.1186/s11671-018-2472-y

**Published:** 2018-02-21

**Authors:** Xiang-Bin Su, Ying Ding, Ben Ma, Ke-Lu Zhang, Ze-Sheng Chen, Jing-Lun Li, Xiao-Ran Cui, Ying-Qiang Xu, Hai-Qiao Ni, Zhi-Chuan Niu

**Affiliations:** 1 National Key Laboratory of Photoelectric Technology and Functional Materials (Culture Base), Institute of Photonics and Photonic Technology, Northwest University, Xi’an, 710069 China; 2 State Key Laboratory for Superlattices and Microstructures, Institute of Semiconductors, Chinese Academy of Sciences, Beijing, 100083 China; 3 College of Materials Science and Opto-Electronic Technology, University of Chinese Academy of Sciences, Beijing, 100083 China; 4 Department of Missile Engineering, Shijiazhuang Campus, Army Engineering University, Shijiazhuang, 050003 China; 5 School of Physics and Nuclear Energy Engineering, Beihang University, Beijing, 100191 China; 6 Wide Bandgap Semiconductor Technology Disciplines State Key Laboratory, Xidian University, Xi’an, 710071 China

**Keywords:** Quantum dot (QD), Annealing, Bimodal size, Molecular beam epitaxy (MBE), Laser

## Abstract

The device characteristics of semiconductor quantum dot lasers have been improved with progress in active layer structures. Self-assembly formed InAs quantum dots grown on GaAs had been intensively promoted in order to achieve quantum dot lasers with superior device performances. In the process of growing high-density InAs/GaAs quantum dots, bimodal size occurs due to large mismatch and other factors. The bimodal size in the InAs/GaAs quantum dot system is eliminated by the method of high-temperature annealing and optimized the in situ annealing temperature. The annealing temperature is taken as the key optimization parameters, and the optimal annealing temperature of 680 °C was obtained. In this process, quantum dot growth temperature, InAs deposition, and arsenic (As) pressure are optimized to improve quantum dot quality and emission wavelength. A 1.3-μm high-performance F-P quantum dot laser with a threshold current density of 110 A/cm^2^ was demonstrated.

## Introduction

Ten years ago, the 1.3-μm quantum dot (QD) laser was developed; however, there has been no distinct development or progress on quantum dot growth since then up till now. The 1.3-μm quantum dot laser has once again become a hot topic of study. It has become one of the strong competitors for the high-speed optical communication local area network (LAN) light source. The high density of quantum dots is an important factor in resulting in low power consumption, high-temperature stability, and high speed. As is well known, the 1.3-μm InAs/GaAs quantum dot laser is expected to exhibit excellent performance at the threshold current, temperature stability, and modulation characteristics due to the three-dimensional quantum confinements [1]. In the last 10 years, a great many laboratories have achieved their aim all over the world, of greatly improving the performance of QD lasers [2–5]. However, bimodal size in InAs/GaAs quantum dot system still exists [6, 7]. The quantum dot quality can be increased if the bimodal size can be eliminated.

InAs/GaAs heterostructures grown by molecular beam epitaxy (MBE) have been paid much attention in order to fabricate low dimensional nanostructures, such as self-assembled QDs due to large lattice (~ 7%) mismatch between InAs layers and GaAs substrate [8]. The growth of InAs on GaAs (001) substrate results in the formation of a three-dimensional (3D) island shape on the InAs with the Stranski-Krastanov (SK) growth mode. The SK growth technique is expected to be a convenient fabrication method of the high-density coherent QDs and is still an open challenge [9, 10]. However, SK QDs have some problems, such as the large inhomogeneous broadening of the QD energy levels and the bimodal size problem [11–15]. For MBE growing high-density quantum dots, the conventional way is to increase the deposition rate of InAs and lower the growth temperature. The purpose of this approach is to reduce the migration rate that can make the formation of the island quickly. However, low-temperature growth may reduce the lattice quality of the epitaxial material. On the other hand, rapid growth can increase the quantum dot density, but it also creates more dislocations. Accordingly, photoluminescence intensity of InAs QDs became weak when we attained a high density of InAs QDs using the conventional approach.

In this letter, single-layer high-temperature annealing can effectively eliminate the defects of the cap material and change the growth direction of dislocations. The size and shape of InAs SK quantum dots show a high degree of uniformity by single-layer annealing that grown on GaAs (001) substrates. There was an increase in the deposition of InAs which improved each QD’s saturation at the same time. The PL spectra of the uniform InAs QDs revealed a narrow linewidth of less than 26 meV. A 1.3-μm InAs/GaAs QD lasers are fabricated which exhibit a lasing threshold current *I*_th_ of 220 mA and a threshold current density of 110 A/cm^2^.

## Material Optimization

In this study, the quantum dot structure is grown on GaAs (001) (N+) substrates in a Veeco Gen 930 MBE system. Annealing temperature has been investigated, and the annealing temperatures for these four samples (N170813, N170824A-N17084C) are 630, 680, 730, and 780 °C, respectively. The growth parameters of quantum dots of these four samples have exactly the same (Table 1).

**Table 1:** Comparison of several types of growth parameters and annealing temperatures

Growth parameters	N170813	N170824A	N170824B	N170824C
Growth T (^°^C)	520	520	520	520
Deposition rate (ML/s)	0.025	0.025	0.025	0.025
Deposition amount (ML)	2.3	2.3	2.3	2.3
III-V ratios (times)	25	25	25	25
Capping In_0.15_GaAs thickness (nm)	5	5	5	5
In_0.15_GaAs rate (ML/s)	0.67	0.67	0.67	0.67
Annealing T (^°^C)	630	680	730	780
Annealing position (nm)	20	20	20	20
Annealing duration (min)	5	5	5	5
PL intensity (a.u.)	23,009.2	26,309.6	18,985.9	9997.8

**Fig. 1:**
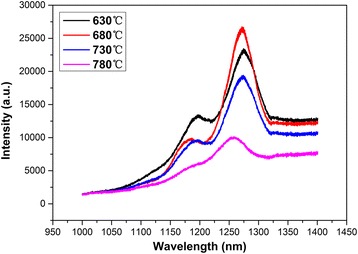
Comparison of photoluminescence (PL) spectra of epitaxial wafers under different annealing temperature

Photoluminescence (PL) measurements were conducted for the four samples. With the increase of annealing temperature, the strongest PL intensity was achieved at the annealing temperature of 680 °C (as shown in Fig. 1). This is because that arsenic (As) and Ga are desorbed as the annealing temperature rises higher. That process can create more defects, and the lattice of InAs quantum dots has changed at high temperature.

**Fig. 2:**
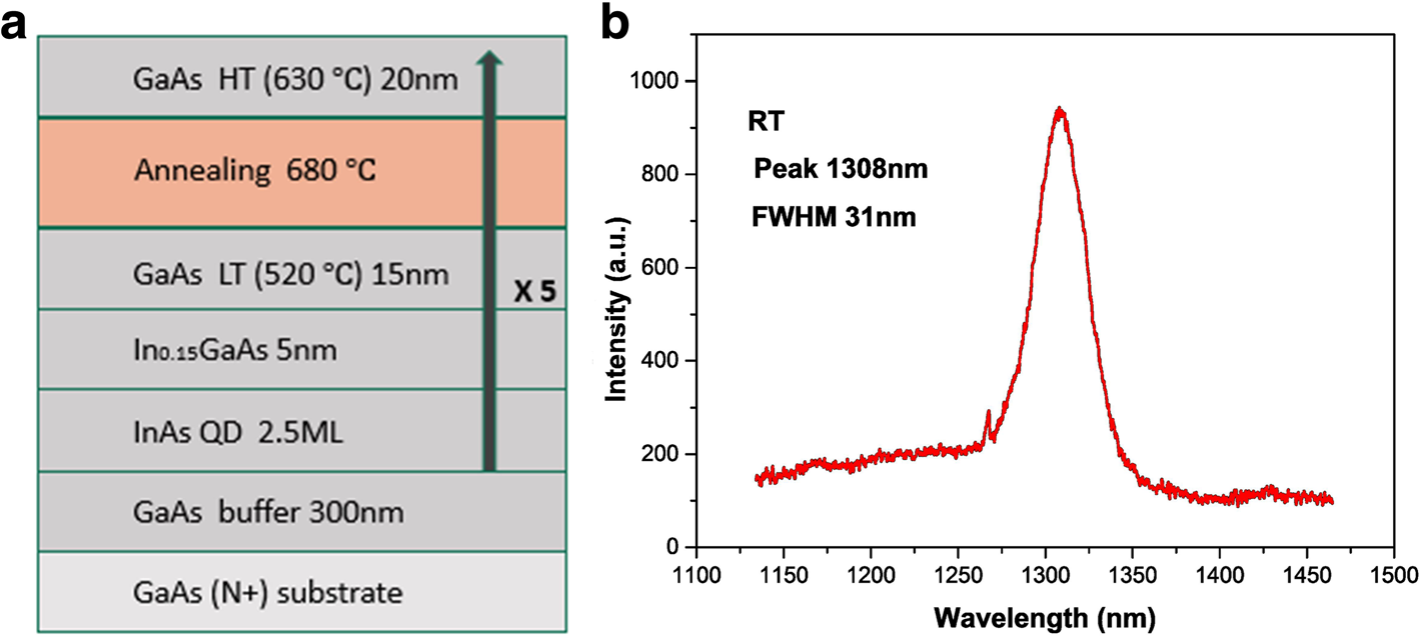
The active region structure and PL spectrum. **a** The structure of the undoped QD laser active region. **b** PL spectrum of the QD laser active region at room temperature (RT). The emission peak is 1305 nm and the FWHM is about 31 nm

Quantum dot laser active area has been optimized at the low arsenic pressure of 4 × 10^− 7^ Torr [16] and low growth rate of 0.025 ML/s. After annealing, we found that the wavelength was less than 1300 nm; therefore, we fine-tuned the growth conditions. A 2.5 monolayer (ML) thick InAs was grown at 520 °C and capped by a 5-nm thick In_0.15_Ga_0.85_As strain-reducing layer at the same temperature. This layer was followed by a 15-nm GaAs layer which deposited at a lower temperature (LT) of 520 °C. Then, we grew the final 20-nm GaAs layer at a higher temperature (HT) of 630 °C (as shown in Fig. 2a).

**Fig. 3:**
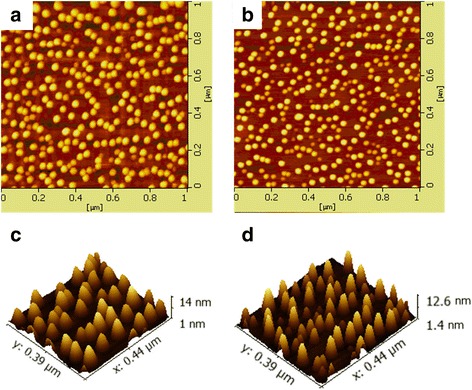
AFM images of the InAs/GaAs QDs. **a** Single layer high-temperature annealing. **b** No annealing. **c** 3D small area size distribution image with high-temperature annealing. **d** 3D small area size distribution image without annealing

The PL spectrum and the atomic force microscopy (AFM) images of the surface of the QDs were measured for the test sample. The emission peak of 1308 nm is due to the ground-state transition, and the full width of half maximum (FWHM) of the peak is about 31 nm (as shown in Fig. 2b). We grew a layer of bare quantum dots on the buried layer of five layers in the test sample to carry out the AFM measurement. The growth conditions are exactly the same as the buried quantum dots described before. The AFM image of the surface of the QDs shows that the QD density of the annealed sample is about 3.2 × 10^10^ cm^− 2^ (as shown in Fig. 3a). The quantum dot has an average height of 8 nm. On the contrary, the unannealed quantum dot sample’s size and distribution are not uniform. Bimodal size can be seen and QD density is about 2.9 × 10^10^ cm^− 2^. The quantum dot has a height of 5–7 nm (as shown in Fig. 3b).

During the epitaxial growth of a 1.3-μm quantum dot laser, the bimodal-size of InAs quantum dots can be well eliminated through the single-layer annealing for the laser active area. Compared with the sample grown without annealing (as shown in Fig. 3c), the sample grown with an annealing temperature at 680 ^°^C (as shown in Fig. 3d) has a higher quantum dot density and a uniform quantum dot size. That can be attributed to the following reasons. At first, GaAs cap layer grows immediately after the growth of InAs quantum dots, so it can only grow at a low temperature, which reduces the lattice quality of GaAs and introduces defects. High-temperature annealing can eliminate defects and can grow high-quality GaAs cap layer used to continue growing InAs quantum dots. In addition, the dislocations are generated during InAs/GaAs heteroepitaxy, in situ single-layer annealing can eliminate dislocation or change the dislocation growth direction and then improve the quality of InAs quantum dots.

## Device Design and Preparation

**Fig. 4:**
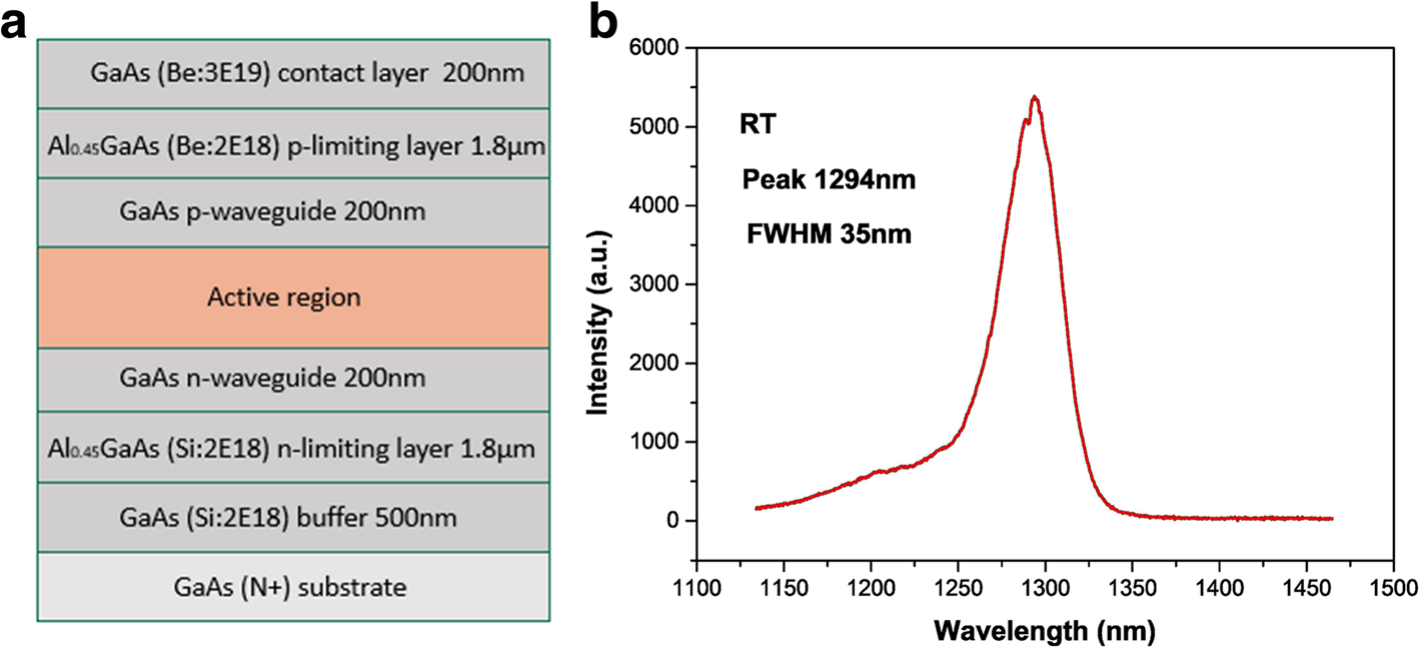
Device structure. **a** 1.3-μm quantum dot F-P broad area laser's epitaxial structure. **b** PL spectrum of the QDs laser's epitaxial structure at RT. The central wavelength is 1294 nm

The laser’s structure consisted of a GaAs layer embedded with five layers of self-assembled InAs QD core layers. The 200-nm n-waveguide layer and p-waveguide layer were grown on top and bottom of the QD structure. The QD active region and waveguide layer were sandwiched by two 1.8-μm p-type (Be: 4E18) and n-type (Si: 2E18) Al_0.45_Ga_0.55_As layers. A 200-nm p+ GaAs (Be: 3E19) layer was deposited for electrical contact (as shown in Fig. 4a).

A small part of the wafer is etched by chemical etching to thin the upper cladding layer with H_3_PO_4_-H_2_O_2_-H_2_O (1:1:4) after the laser epitaxial structure was completed [17, 18]. It can be seen that the PL spectrum of this sample has a central wavelength of 1294 nm (as shown in Fig. 4b). The blue shift of the center wavelength compared to the abovementioned test sample (as shown in Fig. 2a) is due to the high-temperature growth (650 °C) during the growth step of the upper cladding with a growth time longer than 2 h. It also may be from the indium (In) component of the In_0.15_GaAs cap layer’s rock drifts.

**Fig. 5:**
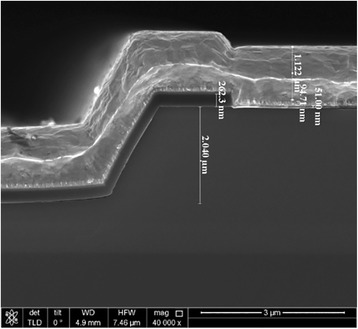
SEM image of the laser's cross section. The F-P broad area laser with a standard laser fabrication process. GaAs/AlGaAs etch depth is about 2-μm. The PECVD formed SiO_2_ is 260 nm

The InAs/GaAs QD laser wafer was coated with photoresist to define the surface pattern. The first edition of photolithography forms a ridge pattern of 100 μm. The ridge waveguide was fabricated by inductively coupled plasma (ICP) etching with an etching depth of 2 μm, followed by Plasma Enhanced Chemical Vapor Deposition (PECVD) in order to form SiO_2_ insulation. In the next step, we made a contact window of 90 μm in width on the ridge for current injection. Then Ti/Pt/Au 51 nm/94.7 nm/1122 nm was deposited as a p-type electrode with magnetron sputtering (as shown in Fig. 5). The wafer is thinned to 120 μm, and a 50-nm thick AuGeNi (80:10:10 wt% alloy) with a 300-nm thick Au layer was deposited on the back of the wafer, using thermal evaporation for n-type electrode [19, 20]. The entire sample was annealed at 460 °C for 10 s in order to form an ohmic contact. During the whole fabrication process, the sample was cleaned sequentially with acetone and isopropyl alcohol and rinsed with deionized water.

**Fig. 6:**
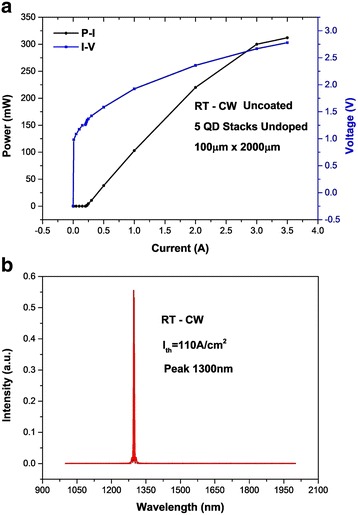
Device measurements. **a** P-I-V curves of a QD laser. **b** The lasing wavelength is 1.3 μm

The electrical and optical properties of the device were measured when the laser was finished. Power-current-voltage (*P*−*I−V*) characteristics of broad area lasers were tested in the continuous wave (CW) at RT. The threshold current density of the laser is 110 A/cm^2^ (as shown in Fig. 6a), and the central wavelength of the lasing spectrum is 1.3 μm (as shown in Fig. 6b). It can be seen from the lasing spectrum that the central wavelength of the laser at room temperature is redshifted because of heating effect of the laser operation. In this study, the laser can continuously lase at room temperature and reach a good threshold current density as well as a good output power without facet coating and undoping in the active region, which indicates the high crystal quality of the laser. The single-layer annealing method has a certain effect on the bimodal size quantum dot system. Deeper level research will be further studied based on this to further improve the density of QDs, in order to achieve a lower threshold current, lower power consumption, higher output power, and high characteristic temperature.

## Conclusions

A series of optimizations of the growth parameters of high-density quantum dots were investigated. The single-layer annealing method was used to successfully suppress the formation of the bimodal-size system of quantum dots. We studied the annealing temperature and annealing layer position in detail. An optimized annealing temperature of 680 °C and a distance from the quantum dot layer of 20 nm were obtained. A threshold current density of 110 A/cm^2^ has been achieved for a 1.3-μm InAs/GaAs QD F-P laser at room temperature and continuous-wave operation with a lasing wavelength of 1.3 μm.

## Abbreviations

AFM: Atomic Force Microscope
Annealing T: Annealing temperature
CW: Continuous wave
F-P: Fabry–Perot
FWHM: Full width at half maximum
Growth T: Growth temperature
HT: High temperature
LT: Low temperature
MBE: Molecular beam epitaxy
PL: Photoluminescence
QD: Quantum dot
RT: Room temperature
SEM: Scanning electron microscope
WPE: Wall plug efficiency
